# Chicoric Acid Is an Antioxidant Molecule That Stimulates AMP Kinase Pathway in L6 Myotubes and Extends Lifespan in *Caenorhabditis elegans*

**DOI:** 10.1371/journal.pone.0078788

**Published:** 2013-11-11

**Authors:** Audrey Schlernitzauer, Catherine Oiry, Raphael Hamad, Simon Galas, Fabienne Cortade, Béatrice Chabi, François Casas, Laurence Pessemesse, Gilles Fouret, Christine Feillet-Coudray, Gérard Cros, Gérard Cabello, Richard Magous, Chantal Wrutniak-Cabello

**Affiliations:** 1 INRA, UMR 866-Dynamique Musculaire et Métabolisme, Montpellier, France; 2 CNRS, UMR 5247-Institut des Biomolécules Max Mousseron (IBMM), Montpellier, France; 3 UFR Pharmacie, Laboratoire de Toxicologie, Montpellier, France; 4 Université Montpellier 1 et 2, Montpellier, France; UAE University, United Arab Emirates

## Abstract

Chicoric acid (CA) is a caffeoyl derivative previously described as having potential anti-diabetic properties. As similarities in cellular mechanism similarities between diabetes and aging have been shown, we explored on L6 myotubes the effect of CA on the modulation of intracellular pathways involved in diabetes and aging. We also determined its influence on lifespan of *Caenorhabditis elegans* worm (*C. elegans*). In L6 myotubes, CA was a potent reactive oxygen species (ROS) scavenger, reducing ROS accumulation under basal as well as oxidative stress conditions. CA also stimulated the AMP-activated kinase (AMPK) pathway and displayed various features associated with AMPK activation: CA (a) enhanced oxidative enzymatic defences through increase in glutathion peroxidase (GPx) and superoxide dismutase (SOD) activities, (b) favoured mitochondria protection against oxidative damage through up-regulation of MnSOD protein expression, (c) increased mitochondrial biogenesis as suggested by increases in complex II and citrate synthase activities, along with up-regulation of PGC-1α mRNA expression and (d) inhibited the insulin/Akt/mTOR pathway. As AMPK stimulators (e.g. the anti-diabetic agent meformin or polyphenols such as epigallocatechingallate or quercetin) were shown to extend lifespan in *C. elegans*, we also determined the effect of CA on the same model. A concentration-dependant lifespan extension was observed with CA (5–100 μM). These data indicate that CA is a potent antioxidant compound activating the AMPK pathway in L6 myotubes. Similarly to other AMPK stimulators, CA is able to extend *C. elegans* lifespan, an effect measurable even at the micromolar range. Future studies will explore CA molecular targets and give new insights about its possible effects on metabolic and aging-related diseases.

## Introduction

In recent years, various polyphenolic molecules have emerged as agents able to modulate cellular pathways involved in metabolic diseases and aging. In particular, similarly to the major antidiabetic drug metformin, resveratrol, quercetin or epigallocatechingallate were found to activate AMP kinase (AMPK), have antidiabetic properties and extend lifespan, mimicking the effects of food restriction or exercise [1].

Also known as a “metabolic master switch”, AMPK is an heterotrimeric Ser/Thr kinase that functions as a cellular energy sensor activated when the cellular AMP/ATP ratio rises [2]. Its activation is mainly induced through Thr172 phosphorylation by the AMPK kinases liver kinase B1 (LKB1) and calcium/calmodulin-dependent protein kinase kinase (CaMKK). Among its numerous metabolic effects, activated AMPK is able to: (a) regulate glycaemia through inhibition of hepatic glucose production and stimulation of glucose uptake [3], [4]; (b) increase fatty acid β-oxidation by inhibition of acetyl-CoA carboxylase (ACC) through Ser-79 phosphorylation, leading to lower malonyl-CoA level and stimulation of carnitine palmitoyltransferase I (CPT1) activity [5], [6]; (c) favour mitochondrial activity through increase in mitochondrial biogenesis and protection against oxidative damage [7], [8] and (d) inhibit the mammalian target of rapamycin (mTOR) pathway [9]–[11]. AMPK is also able to regulate lifespan, as shown in studies performed in mice, *Drosophila melanogaster* fly and *C elegans* worm. In the latter case, it was shown that the knock out of AMPKα2 catalytic subunit gene (*aak-2* gene) reduces in lifespan while overexpression lengthens it [12]. This effect is related to an interaction of AMPK with insulin-like signalling (*Daf* genes) [1], [13]. In addition, AMPK activators were shown to extend lifespan [14].

Aging is also accompanied by muscle mitochondrial dysfunction [15], pointing out some mechanistic similarities between aging and metabolic diseases [16]. In diabetes, the activities of AMPK and mitochondria are impaired by excessive reactive oxygen species (ROS) production while the AMPK activators are able to correct mitochondrial dysfunction [17]. In addition, clinical as well as experimental studies have shown that the AMPK activators metformin [18], [19] or 5-aminoimidazole-4-carboxamide ribonucléotide (AICAR) [20], [21] can prevent diabetes in at risk patients or in the Zucker Diabetic Fatty rat (a model of obesity becoming spontaneously diabetic), respectively. These studies have raised a major interest in the possible anti-diabetic *preventive* effect of agents being able to antagonize ROS accumulation, to stimulate the AMPK pathway and to correct mitochondrial dysfunction.

Among polyphenols, various caffeoyl polyphenolic molecules (e.g. caffeic acid and chlorogenic acid) were shown to display *in vitro* and *in vivo* antidiabetic potential by targeting antioxidant defences, hepatic glucose production, muscle glucose uptake and AMPK activation [22]–[24]. Also, a plant extract, containing both chlorogenic acid and another caffeoyl molecule chicoric acid (dicaffeoyl-tartric acid, CA), increased glucose uptake and insulin secretion in L6 and INS-1 cell lines respectively [24]. CA was also found to induce apoptosis of 3T3-L1preadipocytes through ROS-mediated phosphatidylinositide 3-kinases (PI3K)/protein kinase B (Akt) and mitogen-activated protein kinases (MAPK) signalling pathways, a mechanism that may explain the traditional use of chicoric acid containing plants in obesity [25]. However, although CA was claimed to have some potential to treat metabolic syndrome or diabetes [26], the effects of CA on the AMPK-related pathways have remained unexplored.

In this study, using L6 myotubes, we investigated the effects of CA on oxidative stress, AMPK pathway, mitochondrial activity/biogenesis and Akt/mTOR pathway. Moreover, as these pathways are involved in lifespan modulation, we also determined the effect of CA on *C. elegans* lifespan.

## Materials and Methods

### Studies on L6 muscular cells

#### Materials

Dulbecco's Modified Eagle's Medium (DMEM), Minimal Essential Medium Alpha Medium (αMEM), Dulbecco's Phosphate-Buffered Saline (DPBS), gentamicin, amphotericin B, 2′,7′-dichlorodihydrofluorescein probe (H_2_DCF), Trizol, SuperScript II Reverse Transcriptase and Iblot system were obtained from Invitrogen (CA, USA). Foetal calf serum and horse serum were from Pan Biotech (Bavania, Germany).

Chicoric acid, glucose, palmitate, compound C, and 2-deoxyglucose were purchased from Sigma (Saint Louis, Missouri, USA). Bradford protein assay kit, IQ^TM^ SYBR® Green Supermix and the MJ Mini Thermal cycler were obtained from Bio-Rad (Hercules, CA, USA). Phospho-AMPK (Thr 172), AMPK, phospho-ACC (Ser79), ACC, phospho-Akt (Ser 473), Akt, phospho-mTOR (Ser 2448) and mTOR antibodies were purchased from Cell Signaling Technology (Danvers, MA, USA). α-tubulin was from Sigma (Saint Louis, Missouri, USA). ECL kit was obtained from Thermo scientific (Rockford, IL, USA), PhosphoSafe Extraction Reagent from Novagen (Darmstadt, Germany), and 2-deoxy-D-glucose [1, 2^−3^H] from MP Biomedical (Illkirch, France). Gene Tools software was purchased from Ozyme (St Quentin-en-Yvelines, France).

#### Cell culture

L6 rat myoblasts (ATCC^®^ CRL-1458^™^) were grown in Dulbecco modified Eagle's minimal essential medium (DMEM) supplemented with foetal calf serum (10%), gentamicin (0.05 mg/ml) and amphotericin B (0.5 µg/ml) at 37°C in a humidified incubator (5% CO_2_). L6 myoblats were differentiated into myotubes when 80% of confluence was reached by culturing cells in DMEM supplemented with 2% horse serum instead of 10% foetal calf serum for 7 days. After 7 days of incubation, L6 myotubes were used for the various experiments.

#### Treatment of cell

Chicoric acid (CA) was dissolved in DPBS at 5 mM and added in the culture medium to reach final concentrations from 5 to 100 µM. L6 myotubes were incubated with the different concentrations of CA for 1 hour (short-term effect) or daily for 10 days (chronic effect). During long-term exposure, the culture medium was refreshed daily and newly prepared CA was added each time.

In order to induce oxidative stress, cells were cultured the last 5 days of differenciation in a medium containing 25 mM glucose or 50 µM palmitate.

When compound C was tested, cells were preincubated for 1 hour with 50 µM compound C in DMEM and then incubated for an additional 1 hour with CA.

#### Measurement of enzymatic activities

L6 myotubes grown in 60 mm cell culture dish, were washed and scrapped in DPBS and were centrifugated (2 min, 10 000 rpm). Pellet was incubated 10 minutes in Phosphate buffer (K_2_HPO_4_, 8 mM, pH 7.4) and then lysed by three cycles of freezing/thawing in liquid nitrogen. Protein amount was determined using a Bradford protein assay kit.

Maximal activities of mitochondrial respiratory complexes II, II+III and IV were measured as described previously [27]. Citrate synthase maximal activity was measured according to Srere [28]. Glutathion peroxidase (GPx) and superoxide dismutase (SOD) activities were measured as described respectively by Flohe and Gunzler [29] and Marklund [30]. Enzymatic activities were expressed in mU/mg protein.

#### Measurement of intracellular ROS accumulation

ROS accumulation was determined using H_2_DCF (2′,7′- dichlorodihydrofluorescein) probe. L6 myotubes grown on a 24-well plate were washed with Locke buffer (NaCl 140 mM; KCl 5 mM; MgCl_2_ 1.2 mM; CaCl_2_ 1.8 mM; glucose 10 mM; Hepes 10 mM; Tris-HCl 1M, pH 7.5) and then incubated in the same buffer containing 10 µM H_2_DCF for 20 minutes at 37°C. Fluorescence measurement (λex: 485, λem: 530 nm) was started after addition of the various concentrations of CA using a microplate reader (Synergy2, BiotekFrance, Colmar, France).

#### Western blotting

L6 cells myotubes grown on a 6-well plate were washed with DPBS and lysed in PhosphoSafe Extraction Reagent following the manufacturer's instructions. After centrifugation (10 min, 10 000 g, 4°C), proteins (30 µg) contained in the supernatants were separated by SDS-PAGE electrophoresis and transferred to polyvinylidene difluoride membrane using the Iblot system. After blocking with Tris-buffered saline containing 0.2% Tween 20 (TBST) containing 5% non-fat dry milk for 1 h at room temperature, membrane was incubated overnight at 4°C in TBST, 5% BSA containing the primary antibody. After 3 washes in TBST, membrane was incubated for 1 h with a horseradish peroxidase conjugated secondary antibody. Immunoreactivity was detected with enhanced chemiluminescence detection reagents. Quantification of bands was performed using the Gene Tools software and the intensity values were normalized to tubulin.

#### Quantitative PCR

Total RNA was isolated from L6 myotubes grown in 60 mm cell culture dish with Trizol following the manufacturer's instructions. Reverse transcription reaction was performed with 1 μg total RNA using the SuperScript II Reverse Transcriptase. Real-time PCR analysis was performed using IQ^TM^ SYBR® Green Supermix using the Mini Opticon detection system (Biorad, Hercules, CA, USA). Gene specific primers for PPAR gamma coactivator 1 α (PGC-1α) were designed using Primer Express Software (forward: 5′-TGTGGAACTCTCTGGAAC-3′; reverse: 5′-GCCTTGAAAGGGTTATCTTGG-3′). Normalisation was performed from simultaneous amplification of a RPS9 gene fragment (forward: 5′-GAAGCTGGGTTTGTCGCAAA-3′; reverse: 5′CGGAGCCCATACTCTCCAAT-3′). For both amplifications, after an initial denaturation step for 3 min at 95°C, the thermal cycling conditions were 40 cycles at 95°C for 10 sec and 60°C for 30 sec.

#### Statistical analysis

Statistical significance was assessed using the Student's *t* test.

### *C. elegans* lifespan assays

Wild type Bristol (N2) *C. elegans* worms were provided by the Caenorhabditis Genetic Center: CGC, which is funded by NIH Office of Research Infrastructure Programs (P40 OD010440). Worms where maintained at 15°C and cultured at 20°C as described previously [31]. Five synchronized L4 larvae per well of ELISA 96 plates were fed with HT115 bacteria strain and allowed to grow until death in a final volume of 200 μM of S Medium as previously described [32]. Chicoric acid was added at the indicated final concentrations with FUdr to avoid offspring [33].

Worms were examined every day for touch-provoked movement. Dead worms were scored and worms that displayed extruded internal organs as a result of possible injury during initial transfer were censored.

XLSTAT-life statistical software (Addinsoft, New York, NY, USA) was used to plot survival data by the Kaplan-Meier method and difference between survival curves were calculated by using the LogRank test with 95% confidence. Lifespan experiments were repeated two times with identical results.

## Results

### Influence of CA on ROS accumulation and antioxidant enzymatic defences in L6 myotubes

**Figure 1:**
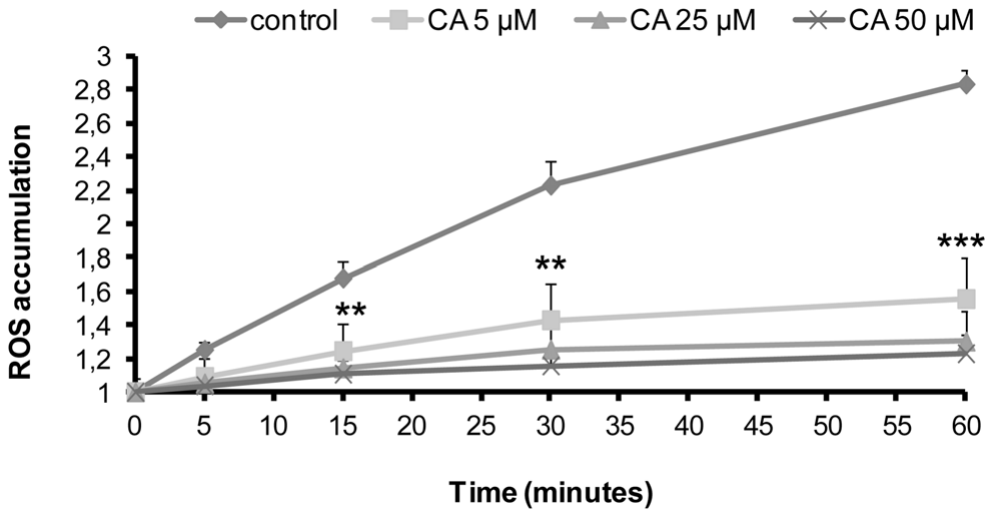
Influence of chicoric acid on ROS accumulation in L6 myotubes. Cells were treated with 5, 25 or 50 µM CA for the indicated times. For each condition, ROS accumulation corresponds to the ratio of the fluorescence measured at the indicated time versus fluorescence at time 0. Values are means ± SEM (n = 3); significantly different from control at ** p<0.01, ***p<0.001.

As illustrated in Fig. 1, basal ROS accumulation time course was assessed in the presence of various concentrations of CA or in its absence (control conditions). In control conditions, cellular ROS accumulation increased gradually. After 60 min incubation, ROS accumulation was concentration-dependently reduced by CA, percentage of inhibition being 69.7%, 83.5% and 87.5% for 5, 25 and 50 µM CA, respectively.

**Figure 2:**
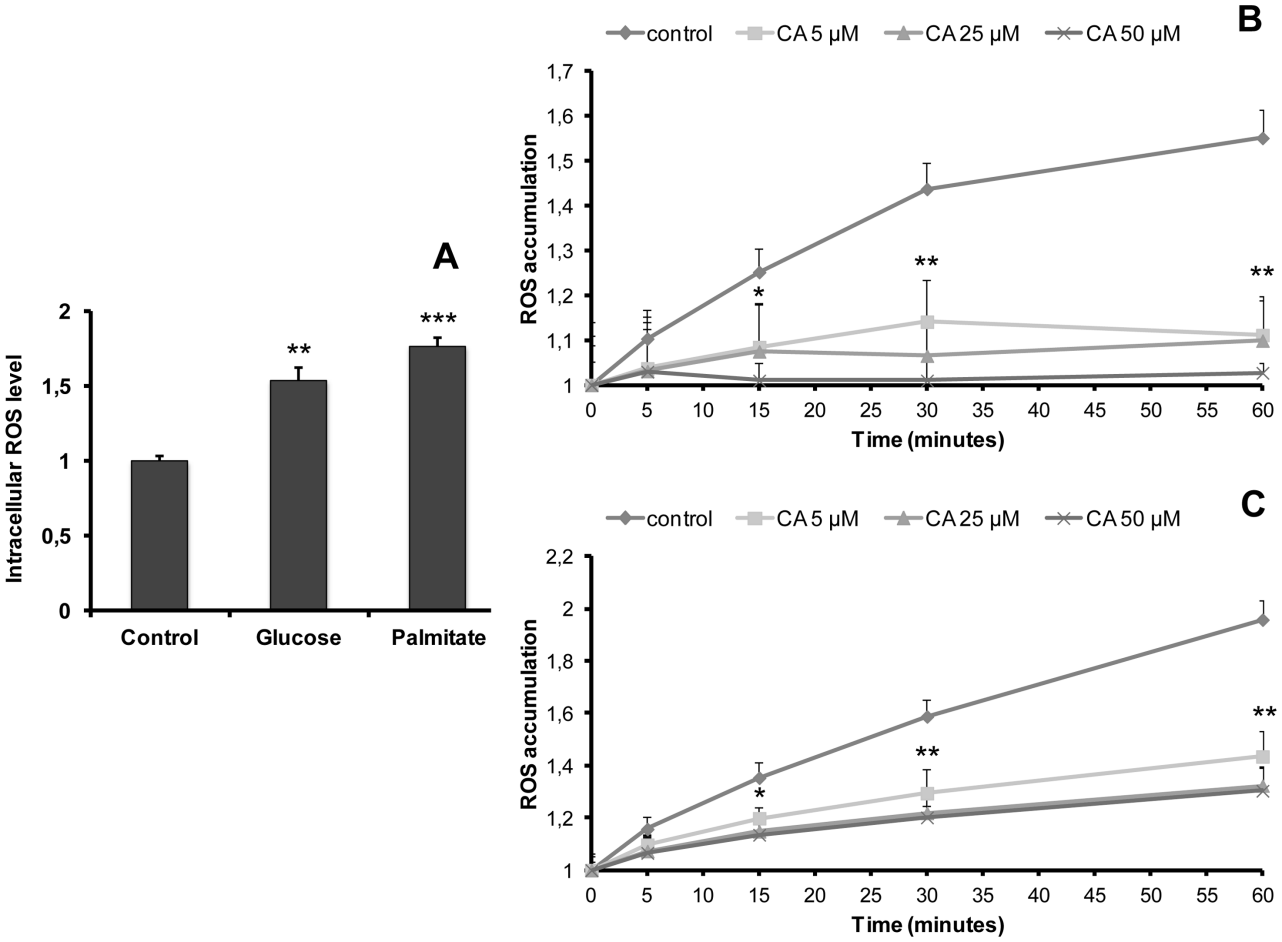
Influence of chicoric acid on ROS accumulation in L6 myotubes after induction of oxidative stress by glucose or palmitate. **A**: Cells were cultured in the absence (control) or presence of glucose (25 mM) or palmitate (50 µM) for 5 days before measurement of intracellular ROS level. Intracellular ROS level is expressed as the ratio of fluorescence determined in control conditions. **Fig**.** 2B–C** illustrate the effect of CA (5, 25 or 50 µM) added for the indicated time after glucose (**B**) or palmitate (**C**) 5-day treatments. ROS accumulation corresponds to the ratio of fluorescence measured at the indicated time versus fluorescence at time 0. Values are means ± SEM (n = 3); significantly different from control at *p<0.05, ** p<0.01, ***p<0.001.

In order to study the antioxidant effect of CA under oxidative stress conditions, cells were cultured for 5 days in a medium containing high glucose (25 mM) or high palmitate (50 µM) concentrations (Fig. 2). As expected, both conditions induced a significant increase in the amount of intracellular ROS (1.6- and 1.9-fold *vs* control, respectively) (Fig. 2A). Figure 2B and 2C illustrate ROS accumulation time-course at the end of the 5-day treatment with glucose (Fig. 2B) or palmitate (Fig. 2C) in the presence of various concentrations of CA or in its absence (control conditions). ROS accumulation appeared to be concentration-dependently reduced. After 60 min incubation, the percentage of inhibition was 79.8%, 82.1% and 95.1% in glucose-treated cells, and 54.6%, 66.2% and 68.3% in palmitate-treated cells for 5, 25 and 50 µM CA, respectively. These data indicate that CA is efficient in preventing ROS accumulation both in basal, high glucose- or high lipid-induced oxidative stress conditions.

**Figure 3:**
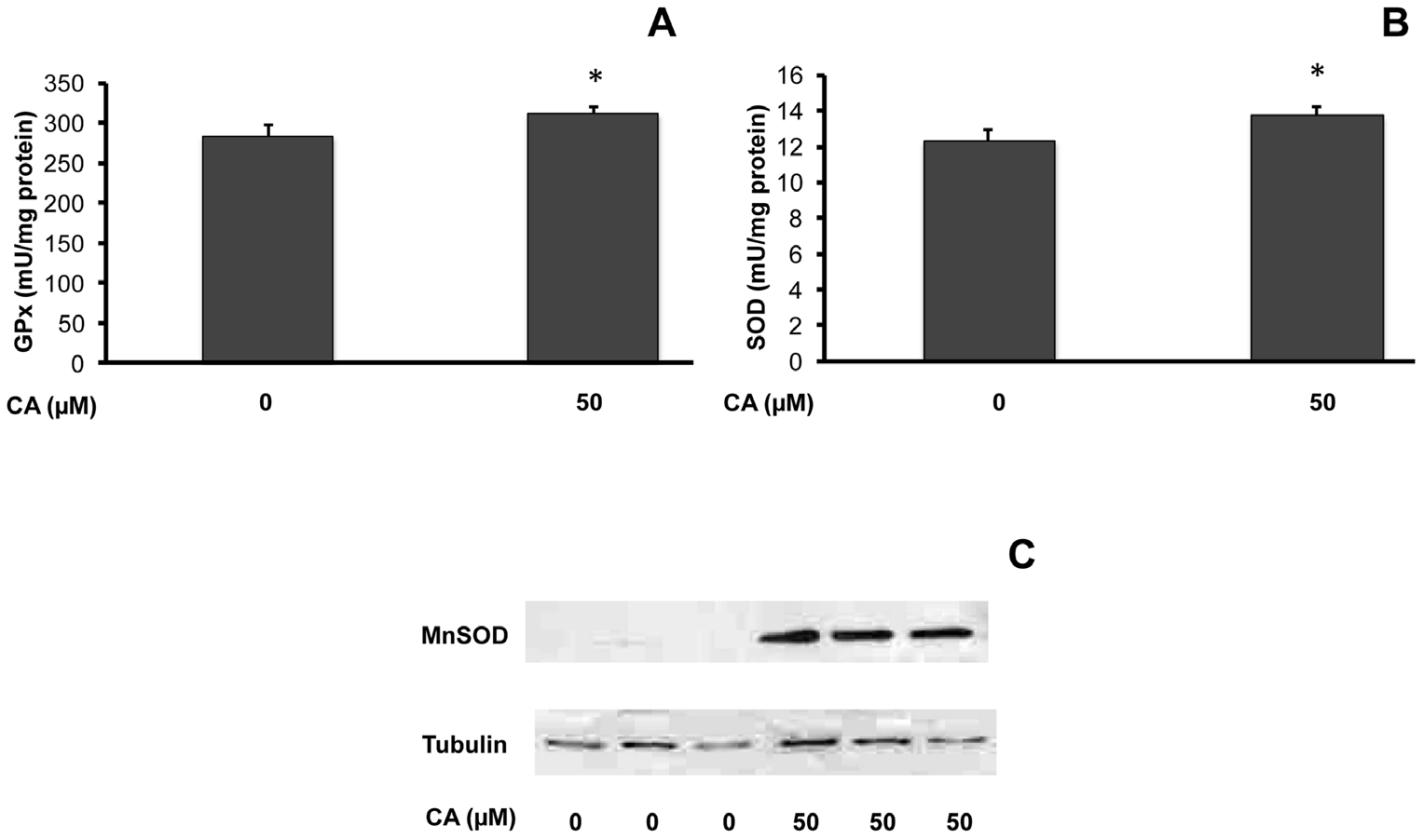
Influence of chicoric acid on antioxidant enzymes in L6 myotubes. Cells were cultured in the presence or absence of CA (50 µM) for 10 days before measurements of the enzymatic activities of GPx (**A**) or SOD (**B**), or analysis of MnSOD protein expression (**C**). Enzymatic activities are expressed as mU/mg protein and indicated as means ± SEM (n = 5); significantly different from control at * p<0.05. Representative immunoblot is shown.

Maximal activity of ROS detoxifying enzymes glutathion peroxidase (GPx) and superoxide dismutase (SOD) and protein expression of mitochondrial SOD (MnSOD) were determined after chronic CA (50 µM) treatment for 10 days (Fig. 3). CA significantly increased GPx (1.10-fold *vs.* control, Fig. 3A) and SOD activities (1.11-fold *vs.* control, Fig. 3B). Strikingly, MnSOD protein levels remained at the brink of detection in control myotubes, but was easily detected in CA treated cells (Fig. 3C), demonstrating the ability of this compound to increase MnSOD protein expression.

These data indicate that CA not only rapidly decreased ROS accumulation but also increased the activity and expression of ROS detoxifying enzymes.

### Influence of CA on mitochondrial activity and PGC-1α expression in L6 myotubes

**Figure 4:**
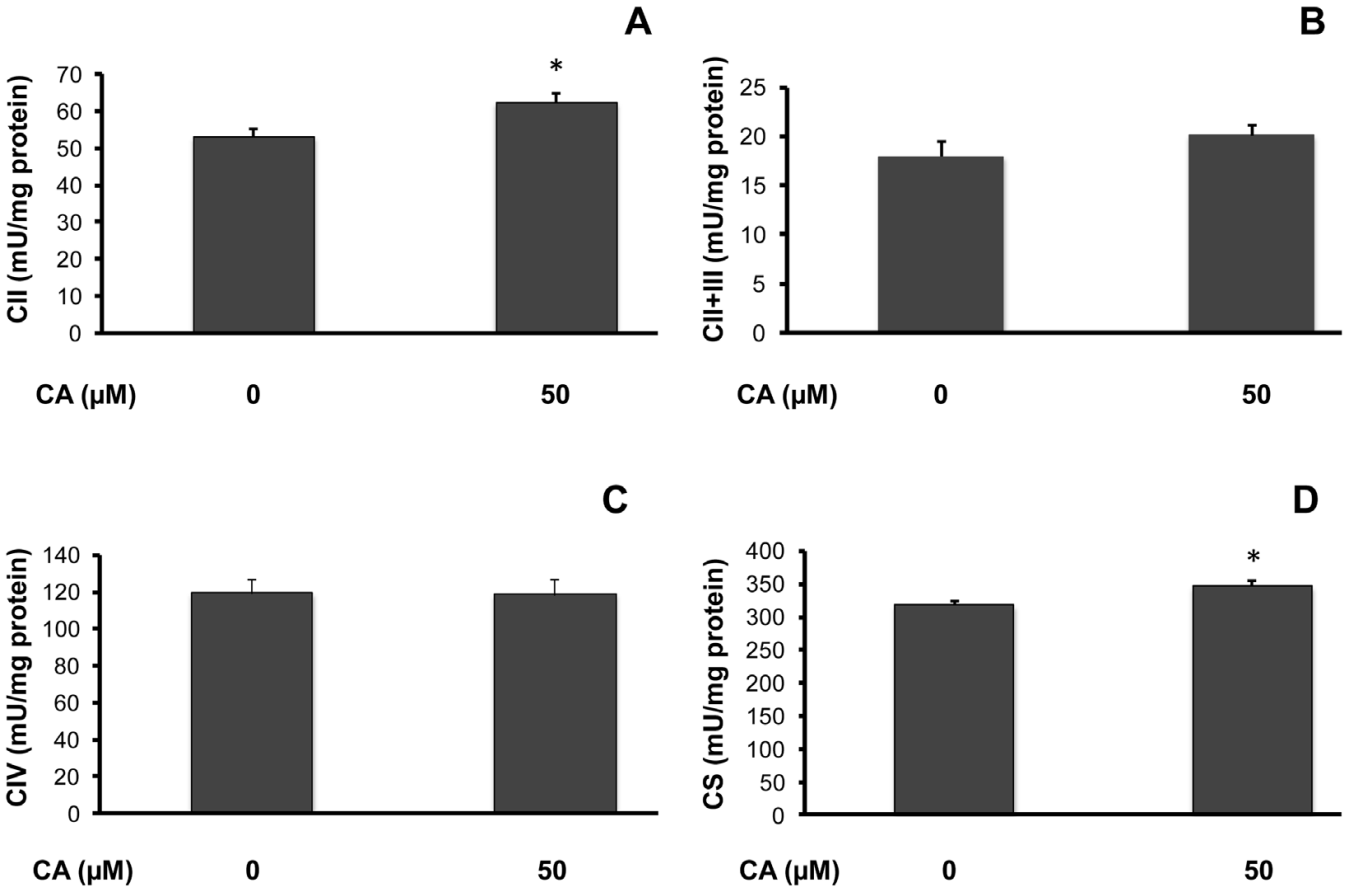
Influence of chicoric acid on mitochondrial activity in L6 myotubes. Cells were cultured in the presence or absence of 50 µM CA for 10 days before measurement of mitochondrial activities. Figure 4 illustrates complex II maximal activity (CII; **A**); complexes II+III maximal activity (CII+III; **B**); complex IV maximal activity (CIV; **C**); and citrate synthase maximal activity (CS; **D**). Enzymatic activities are expressed as mU/mg protein and indicated as means ± SEM (n = 5); significantly different from control at * p<0.05.

It is well known that high amounts of ROS impair respiratory chain activity or mitochondrial biogenesis. We therefore determined the influence of a 10-day CA (50 µM) treatment on mitochondrial activity. As shown in Fig. 4, CA significantly stimulated complex II maximal activity (control: 53.11±1.99, CA: 62.37±2.47 mU/mg protein, a 1.18-fold increase *vs.* control; p<0.05; Fig. 4A) while it did not induce any significant change on complexes II+III (Fig. 4B) and complex IV (Fig. 4C) maximal activity. Furthermore, citrate synthase maximal activity, generally considered as a marker of the mitochondrial mass, was significantly increased by CA (control: 316.94±7.93, CA: 345.30±8.74 mU/mg protein, a 1.09-fold increase *vs.* control; p<0.05; Fig. 4D).

### Influence of CA on PGC-1α mRNA expression in L6 myotubes

**Figure 5:**
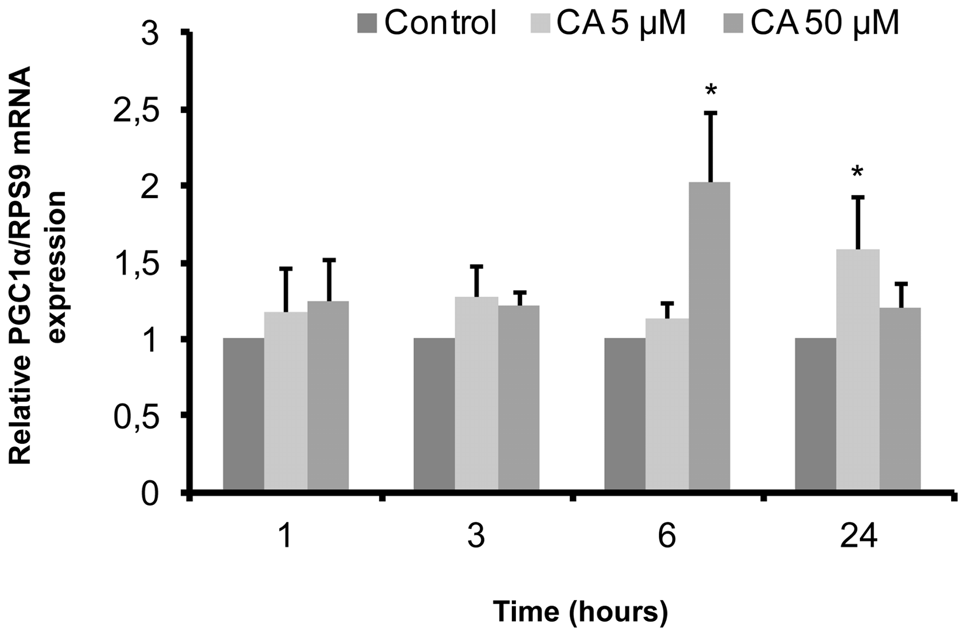
Influence of chicoric acid on PGC-1α mRNA expression in L6 myotubes. Cells were treated with 5 µM or 50 µM CA for the indicated times. PGC-1α mRNA levels were measured by quantitative RT PCR and normalized relatively to RPS9 mRNA expression. Values are means ± SEM (n = 5); significantly different from control of the same time at * p<0.05.

Several studies have shown that PGC-1α is a key regulator of mitochondrial biogenesis and promotes antioxidant enzymes expression [8], [34]–[36]. As we found that CA increased citrate synthase, Gpx and SOD maximal activities, we tested CA influence on PGC-1α expression. As shown in fig. 5, CA induced an increase in PGC-1α mRNA levels, depending on the concentration and duration of incubation. Indeed, PGC-1α mRNA levels increased (2.05-fold) after 6 hours in the presence of CA (50 µM), before returning to basal level after 24 hours. In the presence of 5 µM CA, PGC-1α mRNA levels increased significantly (1.60-fold *vs.* control) after 24 hours.

### Influence of CA on the AMPK/ACC pathway in L6 myotubes

**Figure 6:**
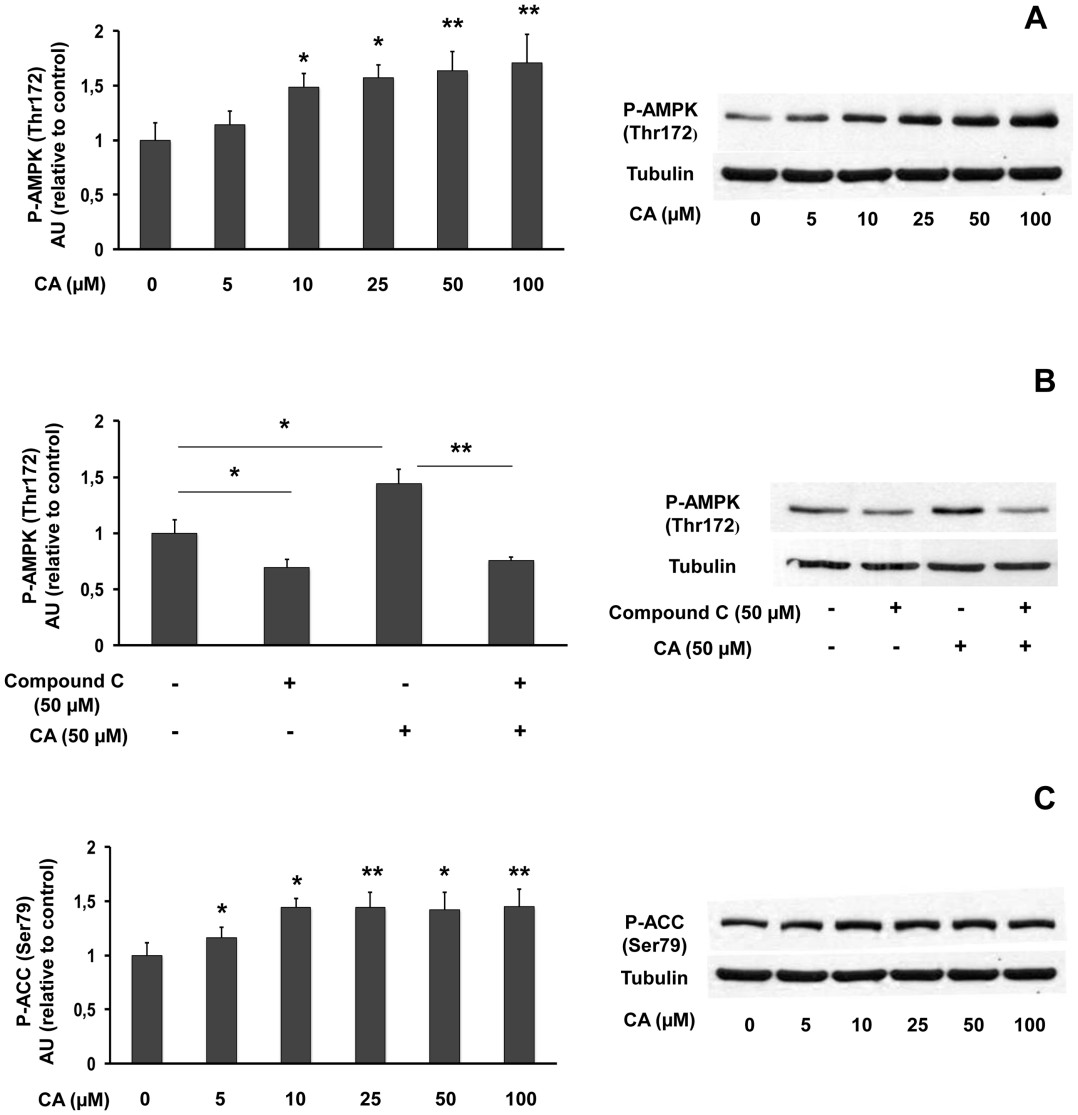
Influence of chicoric acid on AMPK/ACC pathway in L6 myotubes. Cells were treated with the indicated concentrations of CA for 1(Thr172) and tubulin expression (**A**); influence of compound C on AMPK phosphorylation (**B**); phosphorylated ACC (Ser79) and tubulin expression (**C**). Data are expressed relatively to control value (without CA). Values are means ± SEM (n = 5). Significantly different from control at * p<0.05, ** p<0.01. Representative immunoblots are shown.

As it was reported that stimulation of AMPK could increase PGC-1α expression [7], we investigated the influence of a 1-hour CA treatment on the AMPK/ACC pathway (Fig. 6). CA (5–100 µM) did not change the expression of total AMPK (not shown), while inducing a concentration-dependent increase in AMPK phosphorylation (up to 1.71-fold *vs.* control; Fig. 6A). Preincubation of L6 myotubes with compound C (50 µM) (Fig. 6B), a potent inhibitor of AMPK, decreased both basal (31% inhibition as compared to control) and CA-induced AMPK activation (46% inhibition as compared to CA 50 µM). Moreover, CA significantly stimulated ACC phosphorylation even at the lowest concentration used (5 µM: 1.16-fold), with a maximum effect observed for 10 µM (1.45-fold; Fig. 6C). Thus, CA appears to be an activator of the AMPK/ACC pathway at the micromolar range.

### Influence of CA on the Akt and mTOR pathways in L6 myotubes

**Figure 7:**
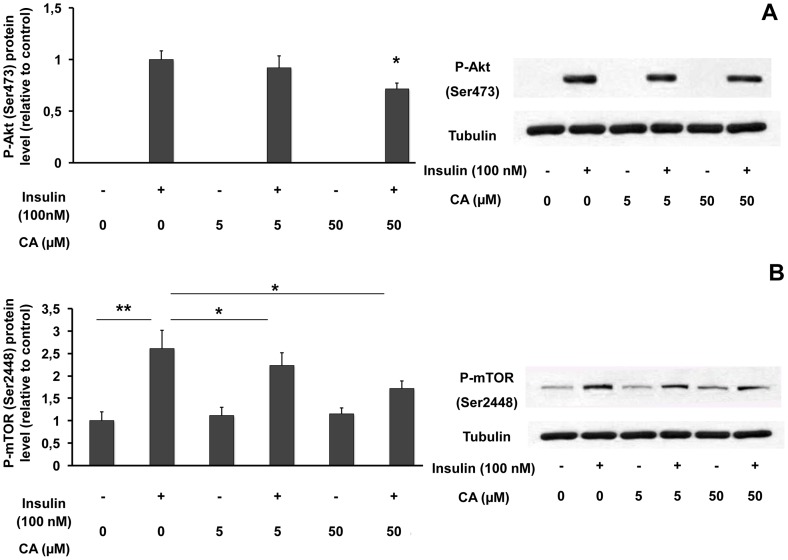
Influence of chicoric acid on Akt and mTOR phosphorylations in L6 myotubes. Cells were treated with 5 or 50 µM CA for 1 hour with or without 100 nM insulin and proteins were extracted for western blot analyses of phosphorylated Akt (Ser473) or tubulin (**A**), phosphorylated mTOR (Ser 2448) or tubulin (**B**). Data are expressed relatively to the value obtained with insulin (without CA) (**A**) or relatively to the value obtained without insulin (without CA) (**B**). Values are means ± SEM (n = 5). * p<0.05, ** p<0.01. Representative immunoblots are shown.

Depending on cellular model, a possible interaction between AMPK and PI3K/Akt pathway has been suggested. So, we analysed the effect of CA on Akt phosphorylation. Figure 7 illustrates the influence of a 1-hour treatment with CA (5, 50 µM), insulin (100 nM) or both, on Akt expression and phosphorylation. Neither insulin nor CA modified total Akt protein levels (not shown). As expected, insulin induced an increase in Akt phosphorylation. Although CA did not influence Akt phosphorylation by itself, 50 µM CA significantly decreased insulin-induced Akt phosphorylation (29% inhibition; Fig. 7A).

We also assessed the influence of CA on mTOR phosphorylation, a downstream target of Akt. Insulin or CA did not significantly influence total mTOR protein levels (not shown). Insulin strongly increased mTOR phosphorylation when compared to control cells. In the absence of insulin, CA did not change mTOR phosphorylation, while it decreased insulin-induced mTOR phosphorylation (5 µM: 14% inhibition; 50 µM: 34% inhibition) (Fig. 7B). Altogether, our results provide evidence that CA inhibits the insulin-dependent stimulation of the Akt/mTOR pathway.

### Influence of CA on C. elegans lifespan

*C. elegans* lifespan can be positively modulated by various types of treatments, notably those aiming at inhibiting the Insulin/Igf-like signalling pathway [37]. Indeed, the reduction of the Insulin/IGF like signalling pathway (in reduction-of-function mutants of the *Daf-2* gene) is susceptible to promote longevity by up to two fold. Longevity is also increased through activation of the AMPK pathway (*aak-2* gene).

**Figure 8:**
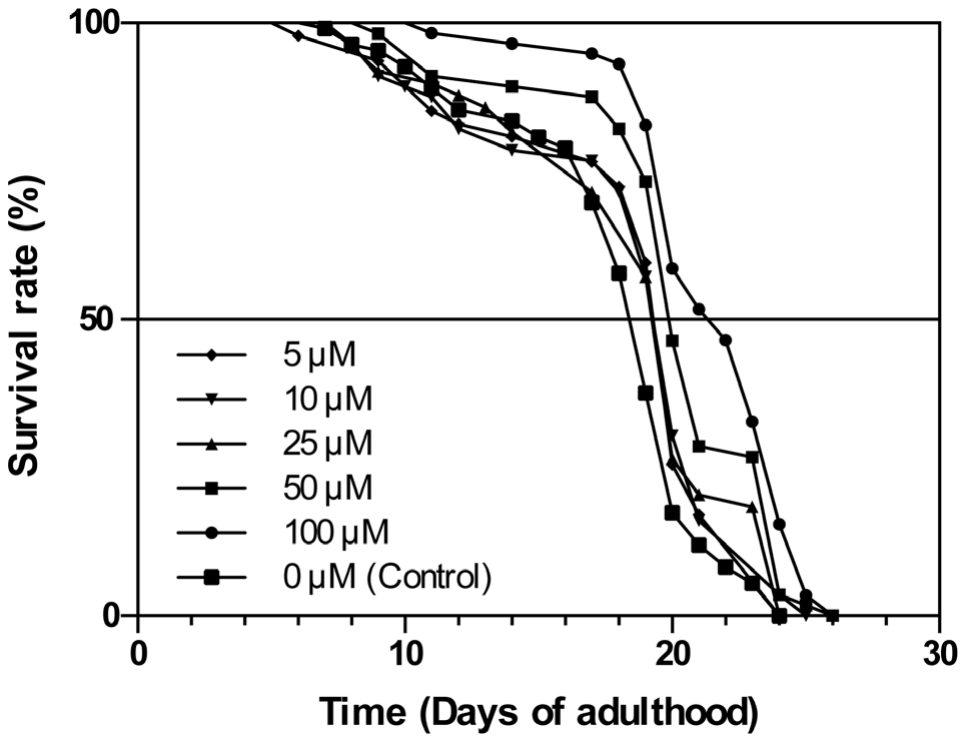
Chicoric acid impact on *C. elegans* lifespan. Wild type (N2) *C. elegans* worm lifespan analysis was performed at 20°C on ELISA 96 plates (see Material and Methods section) at various chicoric acid concentrations as indicated. See Table 1 for corresponding quantitative data and statistical analysis.

We therefore assessed whether CA may modulate *C. elegans* lifespan. As shown in Figure 8 and Table 1, the (N2) wild type worms showed a highly significant longevity extension when treated with 100 µM and 50 µM CA (LogRank p<0.0001), as compared to controls. Moreover, we also observed a less significant, although clearly detectable, average lifespan extension in the presence of a CA concentration as low as 5 µM.

**Table 1:** Chicoric acid-dependent lifespan modulation of *C. elegans*.

Strain	Treatment	50th	Mean lifespan	Max lifespan	Code^b^	Statistics	N
		percentile^a^	days ± SD	days		(KM analysis)^c^	(censored)^d^
N2 (wt)	Control	19	17.94±3.87	24	A	P = 0.0223 (B)	120 (11)
						P = 0.0150 (C)	
						P = 0.0182 (D)	
						P<0.0001 (E)	
						P<0.0001 (F)	
N2 (wt)	5 µM CA	20	18.6±4.46	24	B	P = 0.0223 (A)	60 (13)
						P = 0.8463 (C)	
						P = 0.9821 (D)	
						P = 0.0464 (E)	
						P = 0.0002 (F)	
N2 (wt)	10 µM CA	20	18.52±4.57	25	C	P = 0.0150 (A)	62 (6)
						P = 0.8463 (B)	
						P = 0.8808 (D)	
						P = 0.0588 (E)	
						P = 0.0003 (F)	
N2 (wt)	25 µM CA	20	18.73±4.35	24	D	P = 0.0182 (A)	53 (4)
						P = 0.9821 (B)	
						P = 0.8808 (C)	
						P = 0.0539 (E)	
						P = 0.0003 (F)	
N2 (wt)	50 µM CA	20	20.16±3.76	26	E	P<0.0001 (A)	60 (4)
						P = 0.0464 (B)	
						P = 0.0588 (C)	
						P = 0.0539 (D)	
						P = 0.0657 (F)	
N2 (wt)	100 µM CA	22	21.64±3.76	26	F	P<0.0001 (A)	60 (2)
						P = 0.0002 (B)	
						P = 0.0003 (C)	
						P = 0.0003 (D)	
						P = 0.0657 (E)	

The tabulated data show the average results plotted in Figure 8. Up to two independent trials were done at 20°C with identical results. Worms were fed with the E. coli HT115 strain bacteria (see Material and Methods section). XLSTAT-life statistical software (Addinsoft, New York, NY, USA) was used to plot survival data by the Kaplan Meier method and differences between survival curves calculated using the Log-Rank test with 95% confidence. (a) Represents the 50th percentile (the age when the survival fraction of animals reaches 0.50). (b) Experiment identification code. (c) Probability of being identical to other lifespan experiments given in parentheses. (d) Total death scored (number of censored values).

Moreover, a CA class/effect concentration can be observed on Table 1. We can indeed detect a dichotomy in the impact of CA treatment on maximal *C. elegans* lifespan between 50 and 100 µM CA on one hand (26 days), and 5 to 25 µM CA on the other (24 days), maximal lifespan being similar to that of controls in the latter case.

## Discussion

In this study, using L6 myotubes, we investigated the effects of CA on oxidative stress, AMPK pathway, mitochondrial activity and biogenesis and Akt/mTOR pathway. Moreover, as these pathways are involved in lifespan modulation, we also determined the effect of CA on *C. elegans* lifespan.

Firstly, we report that CA has both short-term influence on ROS accumulation under basal and oxidative stress conditions and long-term effect on the expression of ROS detoxifying enzymes. CA inhibits ROS accumulation in L6 myotubes in basal conditions, or after the induction of oxidative stress by culturing cells in high glucose- or high palmitate- containing medium. These conditions are known to induce insulin signalling pathway defect and insulin resistance [38], [39]. Time-related accumulation of ROS was strongly inhibited even at the lowest CA concentration used (5 µM). This short-term antioxidant activity suggests that it results from a direct interaction with ROS, leading to the reduction of their ability to generate cell damages. We also report that CA increased SOD and GPx maximal activity after a 10-day treatment of cells. This long-term effect may improve the cell ability to manage superoxide anion and H_2_O_2_ detoxification. Furthermore, CA strongly increased the expression of MnSOD, an enzyme localized in mitochondria, which converts the superoxide anion into H_2_O_2_, which is quickly transferred to the cytosol [40]. This effect of CA on MnSOD contribute to protect mitochondria against oxidative damages at the cellular level and may also have some global effect in the organism, as mice overexpressing MnSOD are resistant to oxidative stress and display an increased lifespan [41]. Among other polyphenols, resveratrol (30 µM) also acutely decreases ROS production in HepG2 cells [42] and increases MnSOD expression after long-term (two-weeks) treatment (50 µM) in human lung fibroblasts [43].

Secondly, we report that CA stimulated AMPK phosphorylation and activation. The inhibition of CA-stimulated AMPK phosphorylation by compound C, an inhibitor of AMPK, and the stimulation of ACC phosphorylation by CA are in accordance with an activating effect of CA on the AMPK pathway.

Dysregulation of the AMPK pathway is considered as a contributing factor to the metabolic abnormalities associated with lipid accumulation and insulin resistance [44] found in metabolic syndrome [45], [46] and type 2 diabetes [44], [47]. AMPK, now considered as a main therapeutic target in type 2 diabetes [13] and metabolic syndrome [48], is activated not only by the major anti-diabetic agent metformin, but also by polyphenols with antidiabetic potential such as resveratrol [42], epigallocatechinegallate (EGCG) [49] or quercetin [9]. Activation of AMPK and subsequent ACC phosphorylation reduce the inhibition of CPT1 activity by malonyl CoA, thus favouring fatty acid entry into mitochondria, β-oxidation processes, NADH and FADH2 cofactors production, respiratory chain activity, and ATP production [17]. At the mitochondrial level, β-oxidation preferentially generates FADH2, which provides electrons to complex II [50]. Interestingly, in addition to favouring fatty acid oxidation, CA also increases complex II maximal activity, thus improving mitochondrial ability to efficiently use FADH2 for ATP production.

AMPK activation requires the phosphorylation of Thr172 of a subunit which is mediated by, at least, two distinct upstream kinases, LKB1 and/or CaMKKb. LKB1 activation is dependent on the AMP/ATP ratio while CaMKKb is stimulated by an increase in intracellular Ca2+ level [51 for review]. It was also shown, in cell-free assays, that another kinase, transforming growth factor-β-activated protein kinase-1 (Tak1) could activate AMPK, but the physiological role of Tak1 as an upstream AMPK kinase is poorly documented [52]. On the other hand, deactivation (dephosphorylation) of AMPK is achieved by protein Ser/Thr phosphatases type 2A, (PP2A) or type 2C (PP2C) [53]. Depending on the cellular model and the xenobiotic compound, several different ways for the AMPK activation were described. Metformin, berberine and some polyphenolic compounds, could activate AMPK indirectly via inhibiting mitochondrial ATP production, increasing AMP/ATP ratio leading to AMPK phosphorylation by upstream kinases and/or to a prevention of AMPK dephosphorylation by phosphatases [51], [54]. Caffeoyl derivatives, catechins could activate AMPK via stimulation of LKB1 pathway [55], [56]. Concerning resveratrol, it could activate AMPK via LKB1 pathway at low concentration and via the CaMKKb pathway at high concentration [42], [53]. In our hands, whatever the concentration, CA did not stimulate the activation of AMPK through phosphorylation of the AMPK kinase LKB1 (data not shown). The mechanisms involved in the stimulation of the AMPK/ACC pathway by CA should be further explored.

Interestingly, we have also shown that CA stimulated PGC-1α mRNA expression, in accordance with the fact that stimulation of the AMPK pathway by AICAR was found associated to an increase in PGC-1α expression [7]. Whether there is a causal relationship between both phenomena remains to be determined.

Thirdly, we found that CA could also promote mitochondrial biogenesis, as it increases complex II and citrate synthase maximal activities, as well as PGC-1α expression. PGC-1α is generally described as a major regulator of mitochondrial biogenesis [57]. This effect of CA on PGC-1α expression could, at least partly, explain the increase in mitochondrial mass reflected by the stimulation of citrate synthase activity induced by CA. Resveratrol was also reported to increase PGC-1α expression *in vitro* [58] and *in vivo* [59]. As a decrease in mitochondrial mass and activity has been reported in insulin resistant or type 2 diabetic patients [60], [61], and because mitochondrial deficiency impaired the insulin pathway through cellular fatty acid accumulation [62], it has been proposed that increasing mitochondrial mass in muscle could be an efficient strategy for the prevention of type 2 diabetes [6], [63]. The effect of CA on mitochondrial biogenesis may be beneficial in this regard. In addition, as it has been also shown that PGC-1α up-regulates the expression of genes encoding antioxidant enzymes [8], [36], we can hypothesize that CA effects on GPx and SOD activities and/or expression are mediated through its effect on PGC-1α expression.

Fourthly, we observed that CA decreased the stimulation of Akt and mTOR phosphorylations by insulin, the latter being involved in the positive regulation of protein synthesis and cell growth [64]. It is well known that a decrease in Akt activity inhibits the mTOR/p70S6K pathway. In HT29 colon tumoral cells [10], AMPK activation by synthetic molecules or polyphenolic compounds was shown to activate TSC2, which in turn inhibits TORC1/mTOR and p70S6K. This effect allows the suppression of tumour growth and suggests that these molecules may be used as anti-carcinogenic agents. In breast cancer cells [9], AMPK activation by quercetin abrogated Akt activity while Akt inhibitors alleviated AMPK activities, suggesting a mutual suppressive interaction between Akt and AMPK. In both differentiated neurons and neuroblastoma cells [65], AMPK activation by phenformin or AICAR reduces the activating Ser/Thr phosphorylation of Akt. In agreement with our data, those studies indicate that Akt dephosphorylation often occurs concomitantly with AMPK activation indicating a coordinate reverse regulation of Akt and AMPK.

In skeletal muscle or adipocytes (i.e. GLUT4-expressing cells), activation of Akt [66] or AMPK [67] is generally correlated with an increase in glucose uptake. However, in our hands, CA did not significantly influence glucose uptake either in the presence or the absence of insulin in GLUT4-overexpressing L6 myotubes (data not illustrated). This result can be explained by the fact that simultaneously to a stimulation of AMPK phosphorylation, CA decreases insulin-stimulated Akt activation.

Regarding polyphenolic compounds, controversial effects on glucose uptake were reported. Resveratrol (10–100 µM), which activates AMPK, was shown to be a specific inhibitor of PI3-K/Akt pathway in L6 myotubes and to decrease insulin-induced glucose transport in 3T3 L1 adipocytes [68]. On the other hand, Minakawa et al [69] reported that, in L6 myotubes, resveratrol increased Akt and AMPK phosphorylation and stimulated glucose uptake in the absence of insulin. Another polyphenol, caffeic acid phenethyl ester (CAPE) was shown to enhance insulin-mediated Akt activation and to activate glucose uptake via the AMPK pathway in L6 muscle cells, suggesting that CAPE could directly activates both pathways [43]. However the direct link and the possible interaction between PI3K/Akt and AMPK pathways remain to be confirmed. In the present study, the fact that CA does not influence glucose uptake might be explained by the opposite effects of CA on AMPK (activation) and Akt (inhibition) pathways and/or by the coordinate reverse regulation of Akt and AMPK mentioned above.

In summary, our studies on L6 muscular cells showed that CA is an anti-oxidant that activates AMP kinase and possesses various properties that can be accounted by AMPK activation: (a) it enhanced oxidative enzymatic defence through increase in GPx and SOD activities, (b) favoured mitochondria protection against oxidative damage through upregulation of MnSOD protein expression, (c) increased mitochondrial biogenesis and complex II activity, along with up-regulation of PGC-1α expression and (d) inhibited the insulin/AKT/mTOR pathway.

Interestingly, lifespan is increased by stimulation of AMPK or inhibition of Akt/mTOR pathways and aging associated with oxidative stress and impairment of mitochondrial activity, all features modified by the CA treatment. We therefore determined the effect of CA in *C. elegans* and found that CA treatment was able to extend lifespan in wild type worms, even at the micromolar range, which is at least one order of magnitude lower than any other life-extending agent tested so far [13], [70]–[72]. Noticeably, higher concentrations (50–100 µM) were able to extend both maximal and average lifespan, while lower concentrations (5–25 µM) increased average lifespan only. Maximal lifespan is thought to be controlled by Insulin/Igf-like (*Daf* genes) or AMPK (*aak-2* gene) pathways in *C. elegans* [12], [37]. It is therefore possible that only part of the pathways involved in aging control might be mobilized by lower CA concentrations. In this case, other mechanisms might be involved, such as the redox state (Sir2 pathway *via* NAD^+^−dependent deacetylation) [73] or the mitochondriogenesis control [74] that we also found modified by CA in L6 muscular cells.

Altogether, our data point out the positive modulation on *C. elegans* lifespan by CA, measurable even at a micromolar range. Multiple inter-related pathways that can modulate aging synergistically may explain this highly significant effect of CA. Possible molecular targets of CA are under current investigation and will give new insights about CA effects on aging in *C. elegans* and the possible use of CA in the prevention of aging-related diseases.
